# YTnC2, an improved genetically encoded green calcium indicator based on toadfish troponin C

**DOI:** 10.1002/2211-5463.13702

**Published:** 2023-09-20

**Authors:** Oksana M. Subach, Anna V. Vlaskina, Yulia K. Agapova, Alena Y. Nikolaeva, Anna M. Varizhuk, Oleg V. Podgorny, Kiryl D. Piatkevich, Maxim V. Patrushev, Konstantin M. Boyko, Fedor V. Subach

**Affiliations:** ^1^ Complex of NBICS Technologies National Research Center “Kurchatov Institute” Moscow Russia; ^2^ Bach Institute of Biochemistry Research Centre of Biotechnology of the Russian Academy of Sciences Moscow Russia; ^3^ Federal Research and Clinical Center of Physical‐Chemical Medicine of Federal Medical Biological Agency Moscow Russia; ^4^ Moscow Institute of Physics and Technology Dolgoprudny Russia; ^5^ M.M. Shemyakin and Yu.A. Ovchinnikov Institute of Bioorganic Chemistry RAS Moscow Russia; ^6^ Center for Precision Genome Editing and Genetic Technologies for Biomedicine Pirogov Russian National Research Medical University Moscow Russia; ^7^ Federal Center of Brain Research and Neurotechnologies of Federal Medical Biological Agency Moscow Russia; ^8^ School of Life Sciences Westlake University Hangzhou China; ^9^ Westlake Laboratory of Life Sciences and Biomedicine Hangzhou China

**Keywords:** calcium imaging, crystal structure, genetically encoded calcium indicator, protein engineering, troponin C, YTnC2

## Abstract

Genetically encoded calcium indicators based on truncated troponin C are attractive probes for calcium imaging due to their relatively small molecular size and twofold reduced calcium ion buffering. However, the best‐suited members of this family, YTnC and cNTnC, suffer from low molecular brightness, limited dynamic range, and/or poor sensitivity to calcium transients in neurons. To overcome these limitations, we developed an enhanced version of YTnC, named YTnC2. Compared with YTnC, YTnC2 had 5.7‐fold higher molecular brightness and 6.4‐fold increased dynamic range *in vitro*. YTnC2 was successfully used to reveal calcium transients in the cytosol and in the lumen of mitochondria of both mammalian cells and cultured neurons. Finally, we obtained and analyzed the crystal structure of the fluorescent domain of the YTnC2 mutant.

## Graphical abstract

We present a development of the novel genetically encoded green fluorescent calcium indicator YTnC2 based on toadfish Troponin C. As compared to calmodulin‐based indicators, the YTnC2 indicator offers optimal NTnC‐like design, smaller molecular size, and twofold lower calcium buffering. YTnC2 was successfully applied to monitor calcium transients in the cytosol and mitochondria of neurons. We also determined its crystal structure.

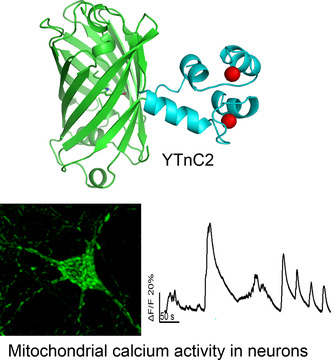

**Abbreviations**
AP: action potential
DIV: day *in vitro*
EDTA: ethylenediaminetetraacetic acid
EGTA: ethylene glycol‐bis(β‐aminoethyl ether)‐*N*,*N*,*N*′,*N*′‐tetraacetic acid
FPLC: fast protein liquid chromatography
FRAP: fluorescence recovery after photobleaching
GECI: genetically encoded calcium indicator
H‐bond: hydrogen bond

Genetically encoded calcium indicators (GECIs) are an indispensable tool for visualization of neuronal activity and calcium signaling in living cells [1]. The most developed and commonly used are green GECIs of GCaMP (jGCaMP8 [2], FGCaMP7 [3]) G‐GECO (G‐GECO1 [4]), and NCaMP (NCaMP7 [5], FNCaMP [6]) families. These GECIs are based on calmodulins and RS20‐ or eNOS‐like variants of peptides. One molecule of calmodulin binds up to four calcium ions. The less common family of Troponin C‐based green GECIs includes the NTnC [7], YTnC [8], iYTnC2 [9], and cNTnC [10] indicators. The truncated version of Troponin C, used in these GECOs, binds two calcium ions per molecule. Hence, as compared to calmodulin‐based GECOs, the Troponin C‐based GECOs offer smaller molecular size and twofold lower calcium ions binding capacity. However, the brightness, dynamic range or sensitivity to action potentials (APs) in neurons for Troponin C‐based GECOs are significantly lower as compared to the calmodulin‐based GECOs. The best members of the Troponin C‐based family, YTnC [8] and cNTnC [10], suffer from low molecular brightness, limited dynamic range, or poor sensitivity to calcium transients in neurons.

To overcome the drawbacks of available Troponin C‐based GECOs, we employed directed a molecular approach to engineer an enhanced version of the YTnC indicator, named YTnC2. As compared to YTnC *in vitro*, the YTnC2 had 5.7‐fold higher molecular brightness, 6.4‐fold increased dynamic range, and similar calcium dissociation rate. In mammalian cells, YTnC2 revealed calcium transients with 3.3‐fold larger dynamics range and 6.8‐fold higher brightness as compared to YTnC. In neurons, YTnC2 demonstrated similar or faster kinetics and 4.6‐ and 1.9‐fold enhanced sensitivity in neurons as compared to the YTnC and cNTnC indicators, respectively.

## Materials and methods

### Cloning of bacterial plasmids, mutagenesis, and library screening

The cloning of bacterial plasmids, mutagenesis, and library screening were performed as described previously [3], using the primers listed in Table S1.

### Protein purification and characterization

Proteins were purified and characterized as described in Ref. [3].

### Mammalian plasmid construction

pAAV‐*CAG*‐NES‐R‐GECO1‐P2A‐NES‐YTnC, pAAV‐*CAG*‐dMito‐YTnC, and mammalian plasmids construction was performed as described in Ref. [8]. In order to construct the pAAV‐*CAG*‐NES‐R‐GECO1‐P2A‐NES‐YTnC2 plasmids, the YTnC2 gene was PCR amplified as BglII‐EcoRI fragment and swapped with the mCherry gene in the pAAV‐*CAG‐*NES‐R‐GECO1‐P2A‐NES‐mCherry vector.

In order to construct the pAAV‐*CAG*‐dMito‐YTnC2 plasmid, the YTnC2 gene was PCR amplified as BglII‐EcoRI fragment and swapped with the mCherry gene in the pAAV‐*CAG‐d*Mito‐mCherry vector. The mammalian plasmids generated in the course of this study are available from the WeKwikGene plasmid repository at Westlake Laboratory, China (https://wekwikgene.resize.rs).

### Mammalian live‐cell imaging

HeLa Kyoto cells (kindly provided by Belousov V.V., Shemyakin‐Ovchinnikov Institute of Bioorganic Chemistry, Moscow) were imaged 24–48 h after the transient lipofectamine transfection before and after 2.5 μm Ionomycin addition using a laser spinning disk Andor XDi Technology Revolution multi‐point confocal system (Andor Technology, Belfast, UK) as previously described [5].

For fluorescence recovery after photobleaching (FRAP) experiments, HeLa Kyoto cells were transfected as described above and imaged using a Leica SP5 STED confocal microscope (Leica‐Microsystems, Bensheim, Germany) and 70% of 488 nm laser power for bleaching (power at 100%—65 mW) during 1000 ms, with the capture settings: 100 ms per frame, 20 and 600 frames before and after bleaching, respectively, resolution—16 × 16 pixels, pixel size—0.38 μm as described earlier [5].

### Isolation, cultivation, and transfection of dissociated neuronal cultures

Neuronal cultures were prepared and cultivated as described in Ref. [3]. Calcium phosphate transfection was performed on DIV 4th. Neuronal cultures plated on 24‐well plates were washed two times with prewarmed MEM (pH 7.1) and left with 500 μL of MEM in incubator at 37 °C and 5% CO_2_. Next, we mixed 1500 ng of plasmid per well (A), 3.1 μL of 2 m CaCl_2_ (0.22 μm filtered) (B), dH_2_O (C) (A + B + C = 25 μL), and 25 μL of 2× HBS. Immediately after mixing, we added 50 μL of DNA precipitate into each well and put neuronal cultures at 37 °C and 5% CO_2_. Twenty minutes after transfection, the cells were incubated with 200–350 μL of HMEM, pH 6.8 (HMEM = MEM + 20 mm HEPES (filtered, pH 7.0)) for 4 min at 37 °C and 5% CO_2_. Washing step with HMEM, pH 6.8 was repeated three times till all precipitate was dissolved. Finally, 500 μL of Neurobasal A medium supplemented with 0.2% B‐27 supplement, 0.5 mm l‐glutamine, 50 U·mL^−1^ penicillin, and 50 U·mL^−1^ streptomycin were added to the neuronal cultures and they were further incubated at 37 °C and 5% CO_2_.

### Visualization of nonspecific mitochondrial calcium neuronal activity

Cultured neurons were prepared from postnatal Day 0 mice (both male and female mice were used) as described previously [11] and co‐transduced at DIV 4–5 with ~ 10^9^ viral particles of rAAV‐DJ‐CAG‐dMito‐YTnC2 and rAAV8‐hSyn‐miRFP (Janelia Farm Viral Core, the rAAV genome titer was determined by dot blot). Functional imaging of transduced neurons was performed using at DIV 12–18 using a Nikon Ti2‐E widefield fluorescence microscope equipped with Spectra III Light Engine (LumenCore, Beaverton, OR, USA), and 20× NA0.75 objective lenses (Nikon, Tokyo, Japan) controlled by NIS‐Elements AR 5.21.00 (Nikon). Structural imaging was performed using on a spinning disk confocal microscope consisting of a Nikon Eclipse Ti‐E inverted microscope and a CSU‐W1 confocal module, with a long‐working‐distance water‐immersion 40× objective (1.15 NA). Fluorescence intensity was measured in mitochondria localized in neuronal somas.

### Protein crystallization

Crystallization of YTnC2‐5 was performed using a robotic crystallization system (Rigaku, Austin, TX, USA) and commercial 96‐well crystallization screens (Hampton Research, Aliso Viejo, CA, USA and Anatrace, Maumee, OH, USA) at 15 °C and 4 °C utilizing the sitting drop vapor diffusion method. The protein concentration was 15 mg·mL^−1^ in the following buffer: 20 mm Tris–HCl, 250 mm NaCl, pH 7.8; 5 mm CaCl_2_. Rod‐like crystals were grown within 6 months in the following conditions: 0.2 m Sodium fluoride, 0.1 m Bis‐tris propane, and 20% PEG 3350 pH 7.5 at 15 °C.

### Data collection, processing, structure solution, and refinement

YTnC2‐5 crystals were briefly soaked in a cryosolution, containing precipitant supplemented with 20% PEG400 immediately prior to diffraction data collection and flash‐frozen in liquid nitrogen. The X‐ray data were collected from a single crystal at 100 K at the beamline BL41XU of the Spring8 synchrotron (Harima, Japan). The data were indexed and integrated in P6_5_ space group using xds program [12]. Scaling was made with aimless [13] (Table S3).

The structure was solved by the molecular replacement method using molrep program [14] and the structure of the genetically encoded green calcium indicator NTnC (PDB ID—5MWC) as an initial model. The refinement of the structure was carried out using the refmac5 program of the ccp4 suite [15]. The visual inspection of electron density maps and the manual rebuilding of the model were carried out using the coot interactive graphics program [16]. The hydrogen atoms in fixed positions were introduced during the refinement together with TLS and twinning option. In the final model, an asymmetric unit contained two independent copies of the protein with chromophore and 214 solvent molecules. Calcium‐binding modules have no electron density in both protein molecules and were not modeled, accordingly.

The visual inspection of the structure was carried out using the coot program and the pymol Molecular Graphics System, Version 1.9.0.0 (Schrödinger, New York, NY, USA).

### Ethical approval and animal care

All methods for animal care and all experimental protocols were approved by the National Research Center ‘Kurchatov Institute’ Committee on Animal Care (protocol No. 1, 7 September 2015) and were in accordance with the Russian Federation Order Requirements N 267 МЗ and the National Institutes of Health Guide for the Care and Use of Laboratory Animals. C57BL/6 mice were used in this study, ages of P0–P2 old. Mice were used without regard to gender.

All animal maintenance and experimental procedures for visualization of nonspecific mitochondrial calcium activity were conducted according to the Westlake University Animal care guidelines, and all animal studies were approved by the Institutional Animal Care and Use Committee (IACUC) of Westlake University, Hangzhou, China under animal protocol #19‐044‐KP.

### Statistics

To estimate the significance of the difference between two values, we used the Mann–Whitney rank sum test and provided *P‐*values calculated for the two‐tailed hypothesis.

## Results and discussion

### Development of the enhanced version of the YTnC indicator, YTnC2

**Fig. 1:**
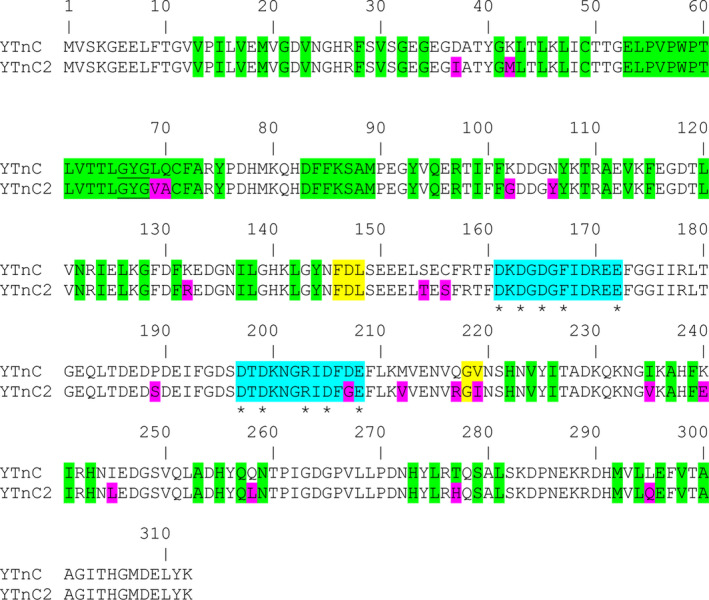
Alignment of the amino acid sequences for the YTnC2 and its progenitor YTnC calcium indicators. Residues from fluorescent part buried in β‐barrel are highlighted with green. Residues that form chromophore are underlined. Calcium ions‐coordinating residues are selected with asterisk according to X‐ray structure of the NTnC indicator [10]. Residues that form Ca^2+^‐binding loops are highlighted in blue. Mutations in YTnC2 related to the original YTnC are highlighted in magenta. Linkers between truncated troponin C and fluorescent domain are in yellow.

**Fig. 2:**
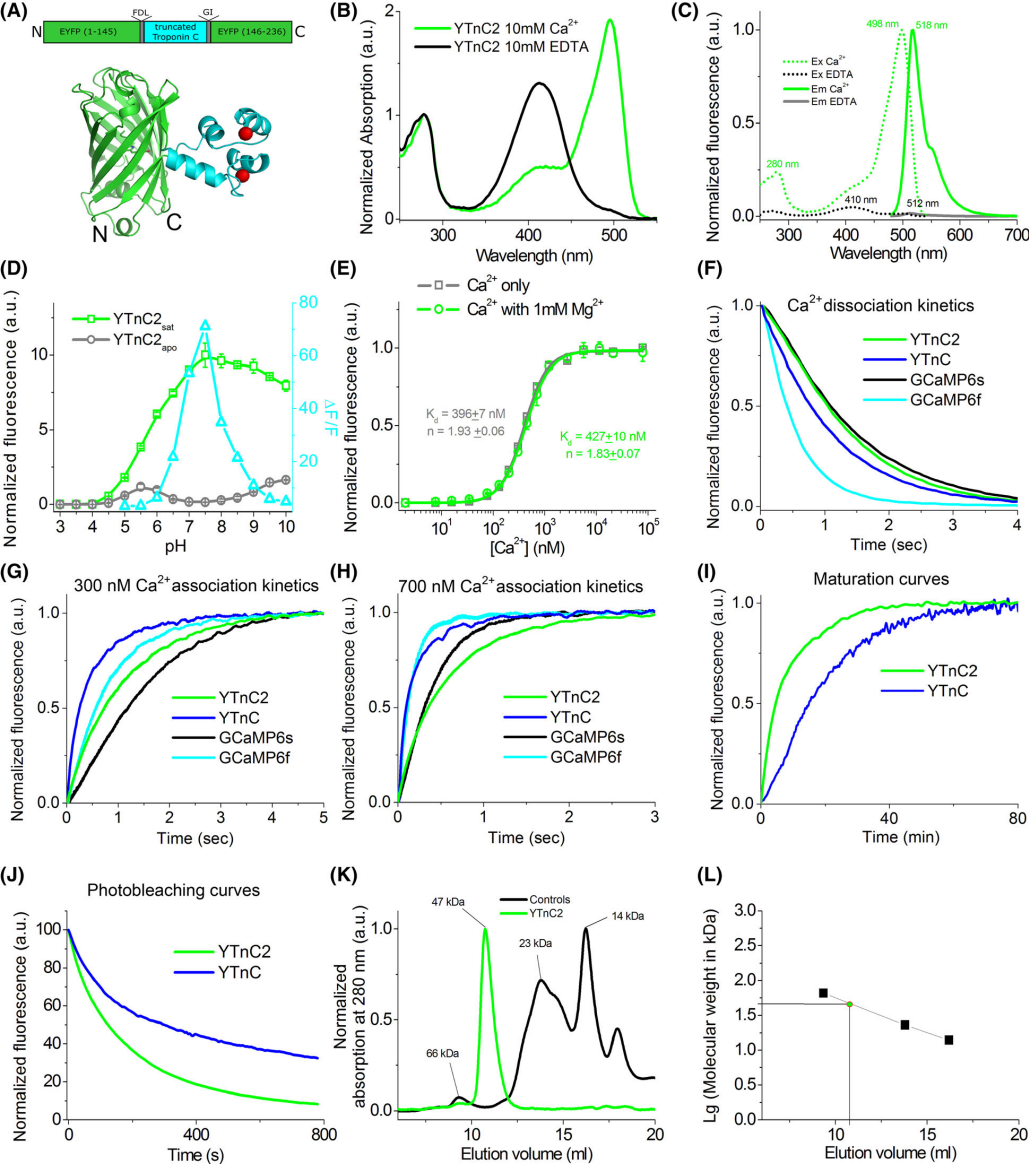
Design and *in vitro* properties of the YTnC2 calcium indicator. (A) A schematic representation of the YTnC2 indicator composition and a cartoon representation of NTnC crystal structure (PDB ID—5MWC) with similar composition. (B) Absorption spectra for YTnC2 in Ca^2+^‐bound (10 mm Ca^2+^) and Ca^2+^‐free (10 mm EDTA) states at pH 7.2. (C) Excitation and emission spectra for YTnC2 in Ca^2+^‐bound (10 mm Ca^2+^) and Ca^2+^‐free (10 mm EDTA) states, pH 7.2. (D) Fluorescence intensity for YTnC2 in Ca^2+^‐bound (10 μm Ca^2+^) and Ca^2+^‐free (10 μm EDTA) states and its dynamic range as a function of pH. (E) Ca^2+^ titration curves for YTnC2 in the absence and in the presence of 1 mm MgCl_2_, pH 7.2. (F) Calcium dissociation kinetics for the YTnC2 and control YTnC, GCaMP6f, and GCaMP6s indicators was acquired at 1000 nm starting Ca^2+^‐free concentration using stopped‐flow fluorimetry. (G, H) Calcium association kinetics for the YTnC2 and control YTnC, GCaMP6f, and GCaMP6s indicators was acquired at 300 (G) and 700 nm (H) starting Ca^2+^‐free concentration using stopped‐flow fluorimetry. (I) Maturation curves for the YTnC2 and YTnC indicators in the presence of 5 mm Ca^2+^, at 37 °C. (J) Photobleaching of YTnC2 and YTnC2 in Ca^2+^‐bound (5 mm Ca^2+^) at pH 7.2, 37 μm protein concentration. The power of 470/40 nm light before 60× objective lens was 1.0 mW·cm^−2^. (K, L) Fast protein liquid chromatography of YTnC2. YTnC2 (37 μm) was eluted in 40 mm Tris–HCl (pH 7.5) and 200 mm NaCl buffer supplemented with 5 mm CaCl_2_ (K). The experimental molecular weight of YTnC2 (47 kDa) was determined from the dependence of logarithm of control molecular weights vs. elution volume (L). The theoretical molecular weight of YTnC2 was 43 kDa. (D–H, J) Three (D–H) and four to nine (J) replicates were averaged for analysis. (D, E) Error bars represent the standard deviation.

To increase brightness and dynamic range of the YTnC indicator, we performed its rational mutagenesis followed by several rounds of random mutagenesis and screening in bacterial cells. To enhance the brightness of the YTnC indicator, we introduced in EYFP‐based fluorescent domain four internal mutations found in the mClover3 bright fluorescent protein to generate YTnC/L69V/Q70A/A73S/T277H mutant (Figs 1 and 2A). Next, we subjected this mutant to nine rounds of random mutagenesis followed by hierarchical screening of bacterial colonies on Petri dishes and crude bacterial lysates. Using Leica fluorescent microscope, on Petri dishes, we picked up about 100 colonies with the largest green fluorescence (excitation 480/40 nm and emission 535/40 nm) contrast before and 24 h after spraying of 100 mm EDTA solution and prepared bacterial streaks. Next, we selected around 15–30 streaks grown on Petri dishes using a Leica microscope to reveal variants with the largest contrast. Finally, we analyzed the most contrast streaks on crude bacterial lysates using a 96‐well plate reader. The best mutant was used as a template for the next round of directed evolution. After nine rounds, we found a mutant, named YTnC2, which had 20 amino acid mutations (Fig. 1 and Fig. S1).

We performed amino acid sequences analysis to understand the potential contributions of the introduced mutations to biochemical properties of the YTnC2 indicator. Nine mutations were external and four mutations were buried inside the β‐barrel of fluorescent domain. Three internal mutations (L69V, Q70A, and T277H) derived from mClover3 protein were suggested to affect the brightness of the YTnC2 indicator. One mutation (V119I) found in the second linker between fluorescent domain and truncated Troponin C could be responsible for the enhanced dynamic range of the indicator. Six mutations were in the calcium‐binding domain. The Q217R mutation was adjacent to the linker and could affect the dynamic range of the indicator. The D207G mutation was close to the calcium coordinating the E208 residue and could affect the affinity of the sensor to calcium ions.

During the final round of the molecular evolution of YTnC2 indicator, we found a variant of YTnC2 with tighter binding of calcium ions. This variant contained five mutations relative to YTnC2 and it was called YTnC2/V69M/G102D/V212M/N223S/L258Q or YTnC2‐5 (Fig. S2). We characterized the main spectral and biophysical properties of YTnC2‐5 *in vitro* (Table S2). According to these data, YTnC2‐5 demonstrated 1.9‐fold tighter calcium binding, but 2.7‐fold slower calcium dissociation kinetics (Table S2). Because of slow calcium dissociation kinetics, we did not characterize YTnC2‐5 in detail and did not choose YTnC2‐5 for further characterization in neurons.

### *In vitro* characterization of the purified YTnC2 indicator

First, we characterized spectral and biochemical properties of the YTnC2 indicator expressed and purified from bacterial cells in solution (Fig. 2 and Table 1). As compared to YTnC, YTnC2 has similar absorption, excitation, and emission maxima (Fig. 2B,C and Table 1). YTnC2 was 5.7‐fold brighter than YTnC in terms of the product of the extinction coefficient and quantum yield (Table 1). The Δ*F*/*F* dynamic range of YTnC2 in the absence and in the presence of 1 mm Mg^2+^ ions (under conditions similar to the cytosol of mammalian cells) was 5.9‐ and 6.4‐fold larger as compared to YTnC (Table 1). Similarly to YTnC, the addition of 1 mm Mg^2+^ ions decreased the dynamic range of YTnC2 by 3.4‐fold (Table 1). In the presence of 1 mm Mg^2+^ ions, the dynamic range of the cNTnC indicator was similar to YTnC2 and the addition of 1 mm Mg^2+^ ions also reduced its dynamic range by twofold [10]. Hence, as compared to YTnC, YTnC2 had similar spectral properties, 5.7‐fold higher molecular brightness, and 6.4‐fold larger dynamic range.

**Table 1:** *In vitro* properties of YTnC2 compared with YTnC.

Properties			YTnC2		YTnC_sat_ ^a^
			apo	sat	
Absorption maxima (nm)			412	496	495 (405)
Emission maxima (nm)			506	518	516 (516)
Quantum yield^b^			0.056 ± 0.004	0.57 ± 0.03	0.19 (0.03)
ε (mm ^−1^·cm^−1^)^c^			42.9 ± 2.4	54.0 ± 2.3	29 ± 3 (20 ± 2)
Brightness vs. EGFP (%)^d^			7.5	97	17 (2)
Δ*F*/*F*	0 mm Mg^2+^		63 ± 2		10.6 ± 0.4
	1 mm Mg^2+^		18.6 ± 0.3		2.9 ± 0.2
p*K*_a_			5.00 ± 0.04 6.29 ± 0.02	5.79 ± 0.10	6.3 ± 0.1
*K*_d_ (nm)^e^	0 mm Mg^2+^		396 ± 7 [*n* = 1.93 ± 0.06]		223 ± 10 [*n* = 1.4 ± 0.1]
	1 mm Mg^2+^		427 ± 10 [*n* = 1.83 ± 0.07]		410 ± 19 [*n* = 1.7 ± 0.2]
*k*_obs_ (s^−1^)^g^	*k*_1_ (contrib., %)	300 nm	1.8 ± 0.1 (31 ± 4)		5.3 ± 0.3 (37.7 ± 0.8)
		700 nm	3.6 ± 0.1 (40 ± 2)		14 ± 3 (60 ± 2)
	*k*_2_ (contrib., %)	300 nm	0.7 ± 0.1 (69 ± 4)		1.12 ± 0.06 (33 ± 2)
		700 nm	1.2 ± 0.1 (60 ± 2)		2.2 ± 0.2 (35 ± 6)
*k*_off_ (s^−1^)^f^			0.81 ± 0.01		0.96 ± 0.01
$t_{1/2}^{\mathrm{off}}$ (s)^h^			0.9 ± 0.1		0.78
Maturation half‐time (min)^i^			4.5		16
Photobleaching half‐time (s)^j^			123 ± 19		299 ± 66
Oligomeric state			Monomer		Monomer

^a^ Values are from Ref. [8]

^b^ Quantum yields (QYs) were determined at pH 7.20. mEGFP (QY = 0.60 [17]) and mTagBFP2 (QY = 0.64 [18]) were used as reference standards for 496‐ and 412‐nm absorbing states, respectively

^c^ Extinction coefficient (ε) was determined by alkaline denaturation; mEGFP in 1× PBS buffer had ε = 53.3 ± 3.6 mm ^−1^·cm^−1^

^d^ Brightness was calculated as a product of the QY and ε

^e^ Hill coefficient is shown in square brackets

^f^ The observed association rates were determined at the indicated Ca^2+^ concentration from association kinetics curves (Fig. 2G,H). GCaMP6f at 300 nm Ca^2+^ concentration had *k*_obs_ value of 1.28 ± 0.01 s^−1^

^g^ *k*_off_ values were estimated from calcium dissociation curves at 1000 nm starting Ca^2+^‐free concentration using mono or double exponential decay fitting with individual exponent contributions shown in the brackets (Fig. 2F). GCaMP6f had *k*_off_ value of 1.89 ± 0.07 s^−1^

^h^ GCaMP6f had *t*_off_ value of 0.37 ± 0.04 s

^i^ EGFP had a maturation half‐time of 14 min

^j^ The power of 470/40 nm light before 60× objective lens was 1.0 mW·cm^−2^.

Next, we characterized the pH stability of YTnC2. As compared to YTnC, the YTnC2 in calcium saturated state had slightly higher pH‐ stability with p*K*_a_ value of 5.79 (Fig. 2D and Table 1). According to previously reported data, cNTnC was less pH stable than YTnC2 [10]. The dynamic ranges of all mentioned indicators depended on the pH (Fig. 2D and Table 1) [10]. The most optimal dynamic range for YTnC2 was achieved at neutral pH from 6.5 to 8.0. Hence, pH changes made an impact on the fluorescence and dynamic range of the YTnC2 indicator.

We further assessed the affinity of YTnC2 to calcium ions. Equilibrium calcium titration experiments revealed calcium affinity for the YTnC2 indicator in the absence and the presence of the 1 mm Mg^2+^ ions of 396 and 427 nm, respectively (Fig. 2E and Table 1). In the presence of the 1 mm Mg^2+^ ions, the affinity of YTnC2 to calcium ions was similar and 1.5‐fold higher than those for YTnC (Table 1) and cNTnC [10], respectively. The presence of Mg^2+^ ions had almost no effect on the calcium dissociation constant for the YTnC2 indicator, in contrast to YTnC and cNTnC, for which Mg^2+^ ions changed their calcium affinity by 1.8‐ and 8‐fold, respectively (Table 1) [10]. Hence, as compared to YTnC, YTnC2 had a similar affinity to calcium ions, which was appropriate for the visualization of calcium transients in the cytosol of mammalian cells and neurons.

Using stopped‐flow fluorimetry, we investigated calcium association–dissociation kinetics for the YTnC2 indicator. Similarly to YTnC, fluorescence of YTnC2 at the binding of 300 and 700 nm calcium ions increased bi‐exponentially, mostly in two steps (Fig. 2G,H and Table 1). Depending on calcium concentration, fast and slow calcium association kinetics for YTnC2 were 1.6–3.9‐fold slower as compared to kinetics for YTnC (Fig. 2G,H and Table 1). Calcium dissociation kinetics of YTnC2 was 1.2‐fold slower than that of YTnC (Fig. 2F and Table 1). Hence, YTnC2 had similar calcium dissociation and 1.6–3.9‐fold slower calcium association kinetics as compared to kinetics of YTnC.

Next, we investigated the maturation rate of YTnC2 at 37 °C. In the presence of calcium ions at 37 °C, YTnC2 maturated 3.5‐fold faster than YTnC (Fig. 2I and Table 1). Under the same conditions, cNTnC matured 17‐fold slower than YTnC2 [10]. Hence, YTnC2 matured 3.5‐ and 17‐fold faster than YTnC and cNTnC indicators.

Next, we studied the photostability of YTnC2 under widefield imaging conditions. Under continuous illumination with a mercury lamp (the power of 470/40 nm light before 60× objective lens was 1.0 mW·cm^−2^) of protein drops in oil, YTnC2 photobleached 2.4‐fold faster than YTnC (Fig. 2J and Table 1). As compared to cNTnC, YTnC2 photobleached 10‐fold faster [10]. Hence, under widefield microscopy conditions YTnC2 was 2.4‐ and 10‐fold less photostable than YTnC and cNTnC, respectively.

The oligomeric state of YTnC2 was further investigated using fast protein liquid chromatography (FPLC). In the presence of calcium ions, YTnC2 eluted as a monomer (Fig. 2K,L). The difference between the experimental and theoretical molecular weight of YTnC2 was only 9%. YTnC and cNTnC were also monomers in FPLC chromatography. Hence, YTnC2 behaved as a monomer in solution.

Overall, as compared to YTnC, monomeric YTnC2 was 5.7‐fold brighter, exhibited 6.4‐fold larger dynamic range, had a similar affinity to calcium ions and calcium dissociation rate, 3.5‐fold faster maturation, but revealed 1.6–3.9‐fold slower calcium association rate and 2.4‐fold poorer photostability.

### Performance of the YTnC2 indicator in HeLa mammalian cells

**Fig. 3:**
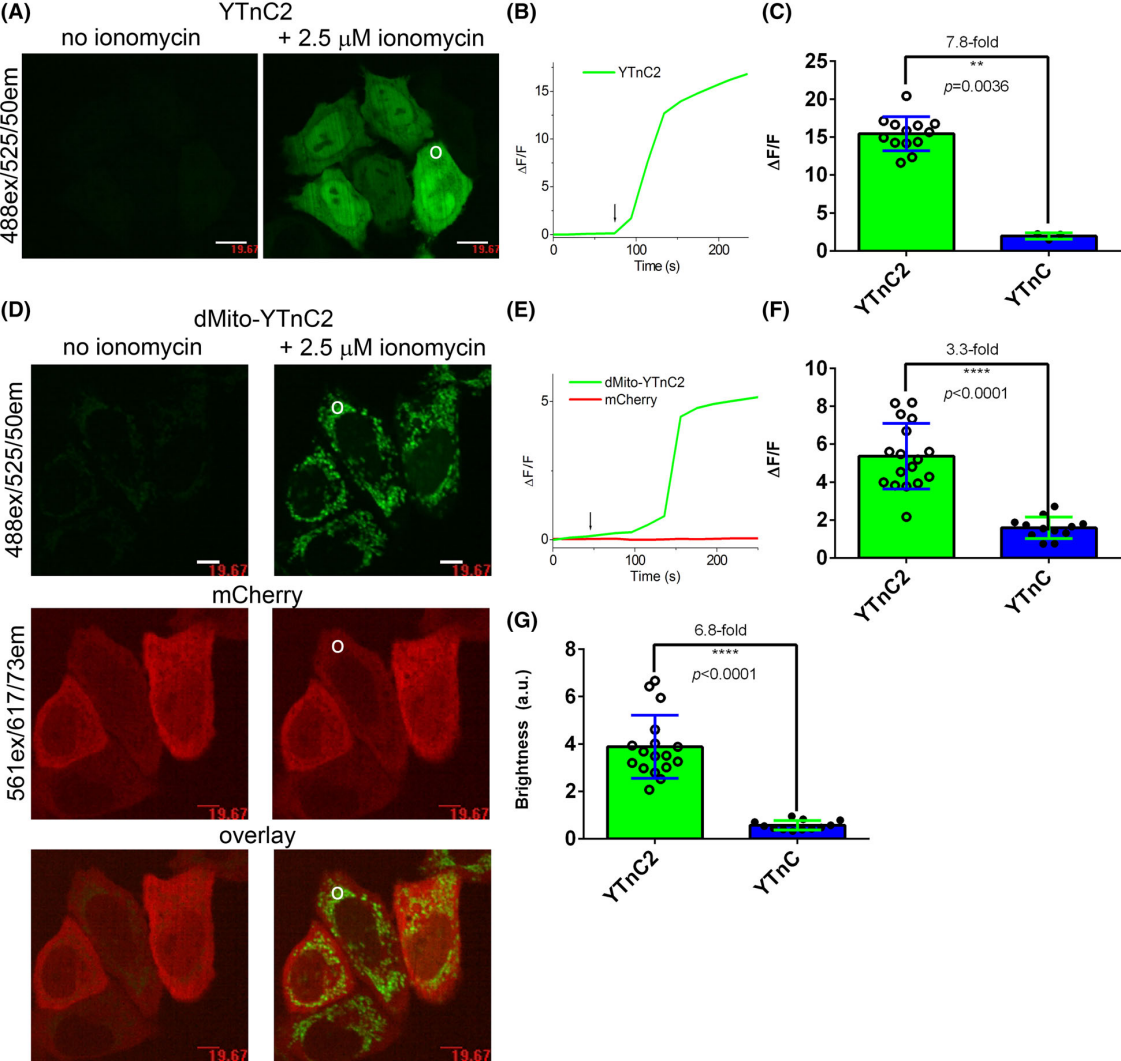
Response of the YTnC2 indicator to Ca^2+^ variations in the cytosol and the lumen of mitochondria of the HeLa cells. (A) Confocal images of HeLa cells expressing green NES‐YTnC2 calcium indicator and mCherry RFP before and after addition of 2.5 μm ionomycin. Scale bars, 20 μm. (B) The graph illustrates changes in green fluorescence of the YTnC2 over time in response to the addition of 2.5 μm of ionomycin (shown by arrow). The changes on the graph correspond to the area indicated with white circle on the panel A. (C) Averaged Δ*F*/*F* responses for the YTnC2 (*n* = 13 cells, two cultures) and YTnC (*n* = 3 cells, one culture) indicators to the addition of the 2.5 μm of ionomycin. (D) Confocal images of HeLa cells co‐expressing green dMito‐YTnC2 calcium indicator and red mCherry RFP before and after addition of 2.5 μm ionomycin. (E) The graph illustrates changes in green fluorescence of the dMito‐YTnC2 and mCherry over time in response to the addition of 2.5 μm of ionomycin (shown by arrow). The changes on the graph correspond to the area indicated with white circle on the panel D. (F) Averaged Δ*F*/*F* responses for the dMito‐YTnC2 (*n* = 17 cells, two cultures) and dMito‐YTnC (*n* = 12 cells, two cultures) indicators to the addition of the 2.5 μm of ionomycin. (G) Averaged maximal brightness of the dMito‐YTnC2 (*n* = 17 cells, two cultures) and dMito‐YTnC (*n* = 12 cells, two cultures) indicators normalized to the red fluorescence of the mCherry RFP. To estimate the significance of the difference between two values, we used the Mann–Whitney rank sum test and provided *P*‐values calculated for the two‐tailed hypothesis. Error bars represent the standard deviation. **P‐value is 0.001–0.01. ****P‐value is < 0.0001.

Next, we assessed the properties of the YTnC2 indicator in the cytosol of HeLa cells using confocal microscopy. Two nuclear export sequences (NES) were attached to the N‐ and C‐termini of the YTnC2 to ensure its localization in the cytosol of the mammalian cells (Fig. S2). When transiently expressed in HeLa cells, YTnC2 was evenly distributed throughout the cytosol and nucleus of the cell (Fig. 3A). Despite the presence of the two NES‐signals, YTnC2 efficiently localized in the nucleus (Fig. 3A). Probably, the small molecular size of YTnC2 allowed it to cross through the nuclear membrane via passive diffusion. YTnC2 visualized calcium elevation in the cytosol of HeLa cells induced by ionomycin addition with Δ*F*/*F* values of 15 ± 2 (Fig. 3A–C), which was 7.8‐fold larger than the Δ*F*/*F* response for YTnC.

**Fig. 4:**
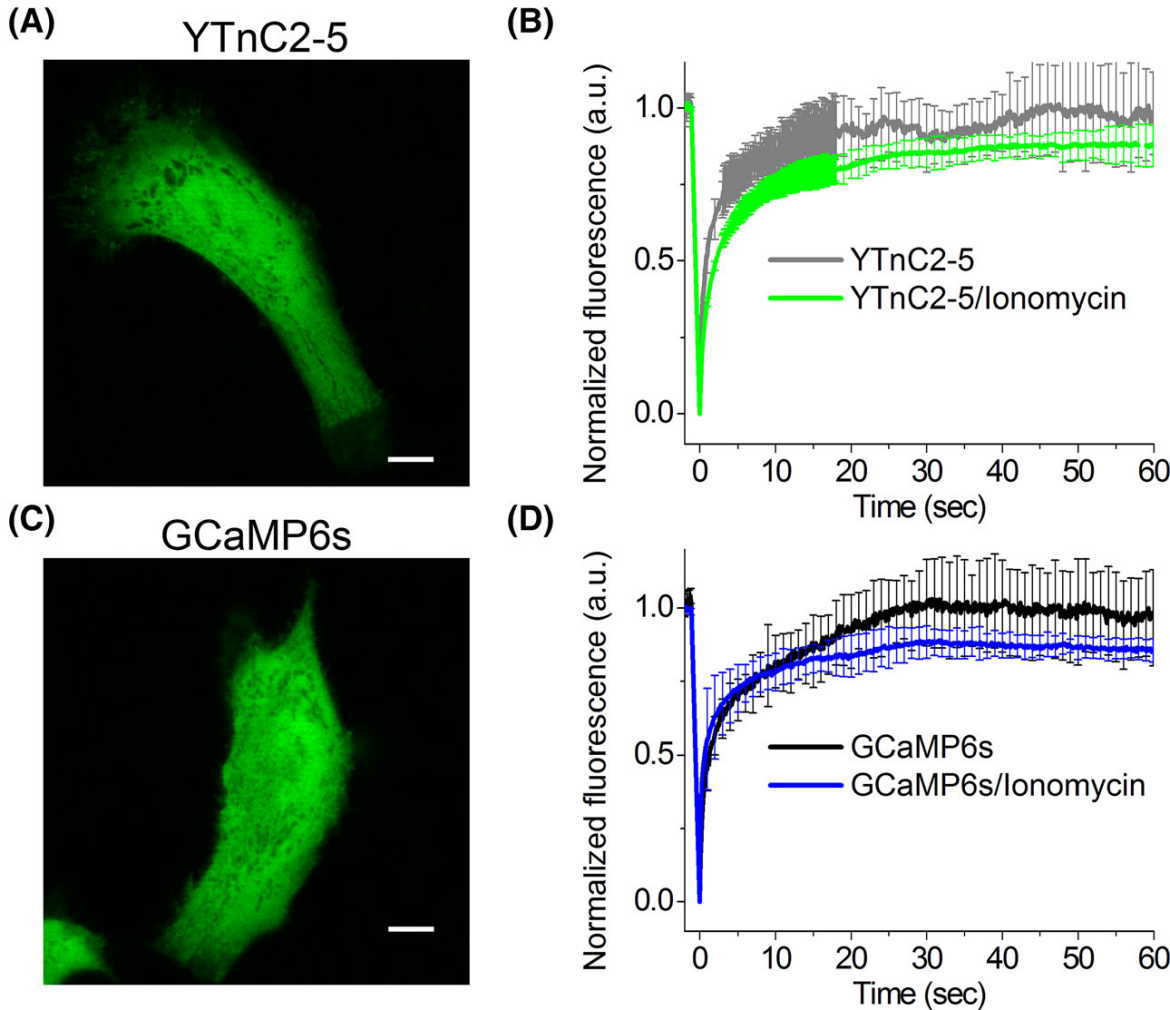
Estimation of mobility of the YTnC2‐5 and GCaMP6s indicator in the cytosol of HeLa cells. (A, C) Example of confocal images of HeLa cells expressing the YTnC2‐5 and GCaMP6s calcium indicators used for the FRAP experiments. Scale bars, 10 μm. (B, D) The graphs illustrate FRAP‐induced changes in green fluorescence of the YTnC2‐5 (B) and control GCaMP6s (D) GECIs at physiological Ca^2+^ concentrations and in response to the 5 μm ionomycin addition for a 60 s time scale. Error bars are standard deviations across five cells.

To characterize the possible interactions of the Troponin C‐based YTnC2 indicator with intracellular components, we conducted FRAP experiments in the cytosol of HeLa cells. The YTnC2‐5 and GCaMP6s indicators were transiently expressed in HeLa cells and imaged using a confocal microscope (Fig. 4A,C). At physiological calcium ions concentrations, the percentages of immobile fractions for YTnC2‐5 and GCaMP6s were 1 ± 17% and 3 ± 15%, respectively (Fig. 4B,D), which were not statistically different from 0%. At elevated ionomycin‐induced calcium ions concentrations, the percentages of immobile fractions for YTnC2‐5 and GCaMP6s increased to 13 ± 4% and 13 ± 5%, respectively (Fig. 4B,D); these percent values were statistically different from 0%. Increased fraction of immobile protein at elevated levels of calcium ions in the cytosol of HeLa cells suggested the existence of some interactions with intracellular environment for both the YTnC2‐5 and GCaMP6s indicators under these conditions. Hence, using FRAP experiments, we did not observe the difference between Troponin C‐based YTnC2‐5 indicator and calmodulin‐based GCaMP6s indicator in terms of interactions with intracellular environment.

Since the YTnC indicator tolerated well N‐terminal fusions [8], we targeted YTnC2 to the lumen of mitochondria via N‐terminal fusion with mitochondrial presequence of human cytochrome c oxidase subunit VIII (Fig. S2) and studied its brightness and dynamic range using confocal microscopy. According to confocal images of transiently transfected HeLa cells, dMito‐YTnC2 fusion localized in mitochondria of HeLa cells (Fig. 3D and Fig. S3); mCherry RFP was co‐expressed in the same cells for normalization of intracellular brightness. The addition of 2.5 μm ionomycin resulted in an increase in green fluorescence of dMito‐YTnC2 and control dMito‐YTnC fusions with Δ*F*/*F* values of 5.4 ± 1.7 and 1.6 ± 0.6, respectively (Fig. 3D–F). In response to the addition of the 10 μm thapsigargin (an inhibitor of the sarco/endoplasmic reticulum Ca^2+^ ATPase (SERCA)), the dMito‐YTnC2 indicator increased its fluorescence by 3.5 ± 1.3 fold; this increase was 1.4‐fold larger as compared to the respective response of 2.5 ± 1.1 for the control YTnC indicator (Fig. S3). The maximal brightness of dMito‐YTnC2 after ionomycin and thapsigargin addition normalized to the red fluorescence of the mCherry RFP was 6.8‐ and 5.6‐fold larger as compared to the brightness of the dMito‐YTnC, respectively (Fig. 3G and Fig. S3). The difference in brightness between YTnC2 and YTnC in mitochondria practically coincided with the difference between them in molecular brightness *in vitro*. We observed a lag in the rise in the Ca^2+^ transient when the cells were stimulated with ionomycin when YTnC2 was targeted to the lumen of mitochondria (Fig. 3E) as compared to that in the cytosol (Fig. 3B). Similarly, mitochondrial rise of calcium concentration was shown to be lagged behind the cytosolic calcium increase in mammalian cells using D1GO‐Cam cameleon calcium indicator [19]. The observed delay in induced calcium transients between two organelles was attributed to the lower calcium affinity and kinetics of the mitochondrial calcium uptake machinery. Hence, Δ*F*/*F* dynamic range and brightness of dMito‐YTnC2 fusion localized in the lumen of mitochondria of HeLa cells were 1.4–3.3‐ and 5.6–6.8‐fold larger as compared to dMito‐YTnC fusion, respectively (Fig. 3F,G and Fig. S3).

### Visualization of activity of neuronal cultures using the YTnC2 indicator

**Fig. 5:**
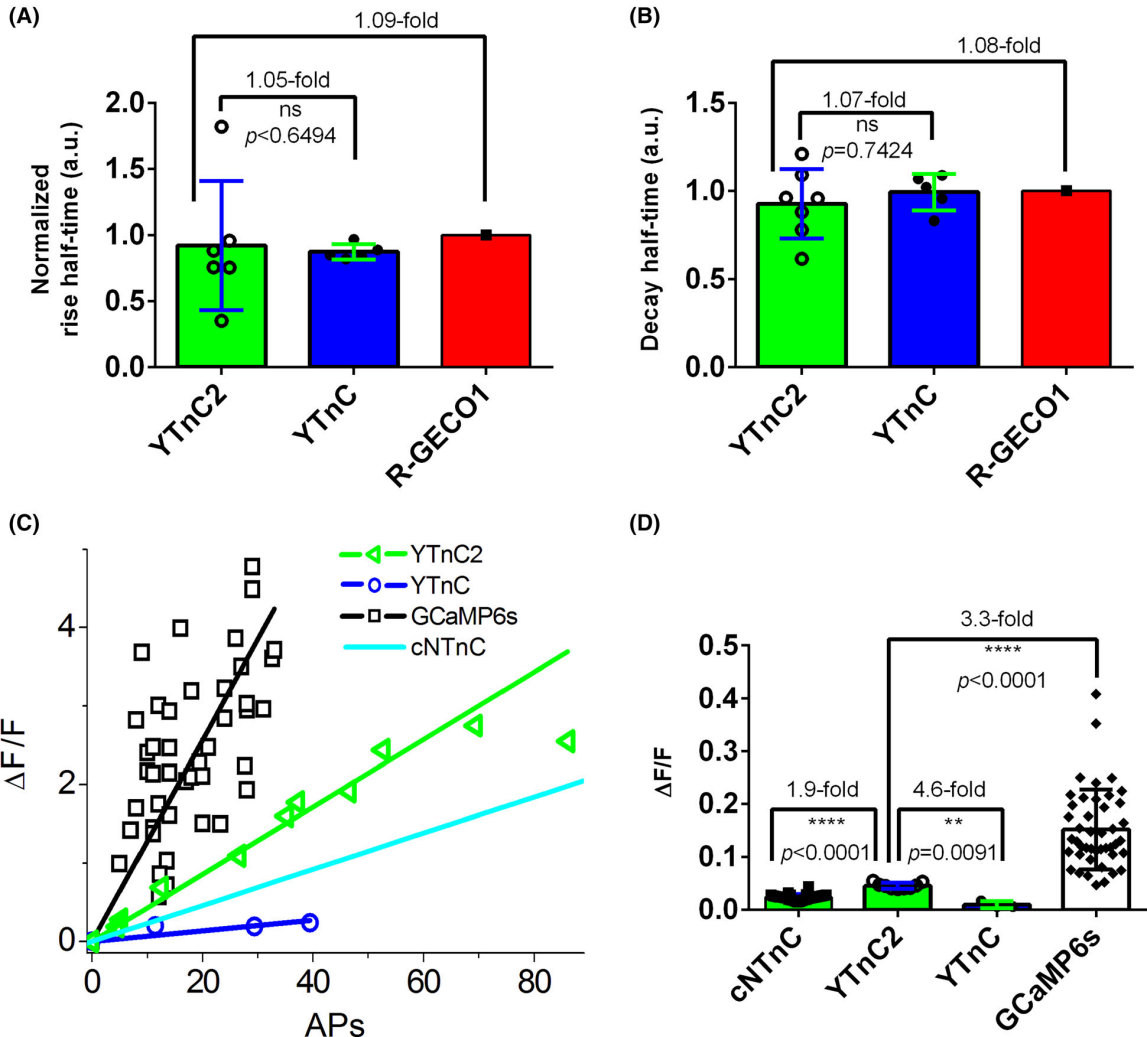
Visualization of neuronal activity using the YTnC2 indicator. Neuronal cultures co‐expressing the NES‐R‐GECO1 and NES‐YTnC2 or NES‐YTnC indicators were imaged and stimulated on DIV 7th. Neuronal cultures were transfected on DIV 4th by calcium phosphate method using plasmid carrying *CAG*‐NES‐RGECO1‐P2A‐NES‐YTnC2 or *CAG*‐NES‐RGECO1‐P2A‐NES‐YTnC. P2A is a self‐cleavable peptide. (A, B) Averaged rise (A) and decay half‐times (B) for YTnC (*n* = 5, two cultures) and YTnC2 (*n* = 6–7, two cultures) were normalized to the respective half‐times for R‐GECO1 in the same cell. (C) The dependence of Δ*F*/*F* responses for the YTnC2 and YTnC indicators vs. the number of APs. A number of APs were determined according to the Δ*F*/*F* response of the R‐GECO1 indicator (0.04 per 1 AP) co‐expressed in the same cell and assuming linearity of the response of R‐GECO1 in the examined AP range. The dependences of Δ*F*/*F* responses on APs for R‐GECO1, G‐CaMP6s, and cNTnC were added to compare the results with previous works [3, 10]. (D) The Δ*F*/*F* responses per one AP for YTnC2 (*n* = 9, one culture) and YTnC (*n* = 3, one culture) were calculated according to the Δ*F*/*F* response of R‐GECO1 (0.04 per 1 AP [20]) in the same cell. For comparison, the Δ*F*/*F* responses per one AP for cNTnC and G‐CaMP6s were added from previous works [3, 10]. (A, B, D) Error bars are the standard deviations. Ns, not significant. **P‐value is 0.001–0.01. ****P‐value is < 0.0001. To estimate the significance of the difference between two values, we used the Mann–Whitney rank sum test and provided *P* values calculated for the two‐tailed hypothesis.

To characterize YTnC2 in neuronal cultures, we monitored its kinetics and Δ*F*/*F* dynamic range in neuronal cultures during their nonspecific and stimulated activity. On day *in vitro* (DIV) 4th, neuronal cultures were transfected by pAAV‐*CAG*‐NES‐YTnC2‐P2A‐NES‐R‐GECO1 or pAAV‐*CAG*‐NES‐YTnC2‐P2A‐NES‐R‐GECO1 plasmids using calcium phosphate transfection method. On DIV 7th, we recorded the calcium activity of the cultures using a confocal microscope. According to nonspecific activity of the neuronal cultures, the rise and decay half‐times were similar among YTnC2, YTnC, and R‐GECO1 indicators (Fig. 5A,B). Since the decay half‐time for cNTnC was 2.5‐fold slower than that for R‐GECO1 [10], consequently decay dynamics for YTnC2 was 2.7‐fold faster. Stimulation of neuronal cultures with electric field revealed that YTnC2 demonstrated linear dependence of its Δ*F*/*F* response over a number of APs in the range of 0–70 APs (Fig. 5C). Averaged Δ*F*/*F* response per 1 AP for YTnC2 was 4.6‐ and 1.9‐fold larger than those for YTnC and cNTnC, respectively (Fig. 5D). Averaged Δ*F*/*F* response per 1 AP for YTnC2 was still 3.3‐fold smaller than the same characteristics for standard GCaMP6s indicator (Fig. 5D). Hence, as compared to the YTnC and cNTnC indicators, YTnC2 visualized neuronal calcium activity with 4.6‐ and 1.9‐fold higher sensitivity and similar or faster kinetics, respectively, but it was still 3.3‐fold less sensitive than calmodulin‐based GCaMP6s GECI.

### Visualization of mitochondrial calcium activity of neuronal cultures using the dMito‐YTnC2 indicator

**Fig. 6:**
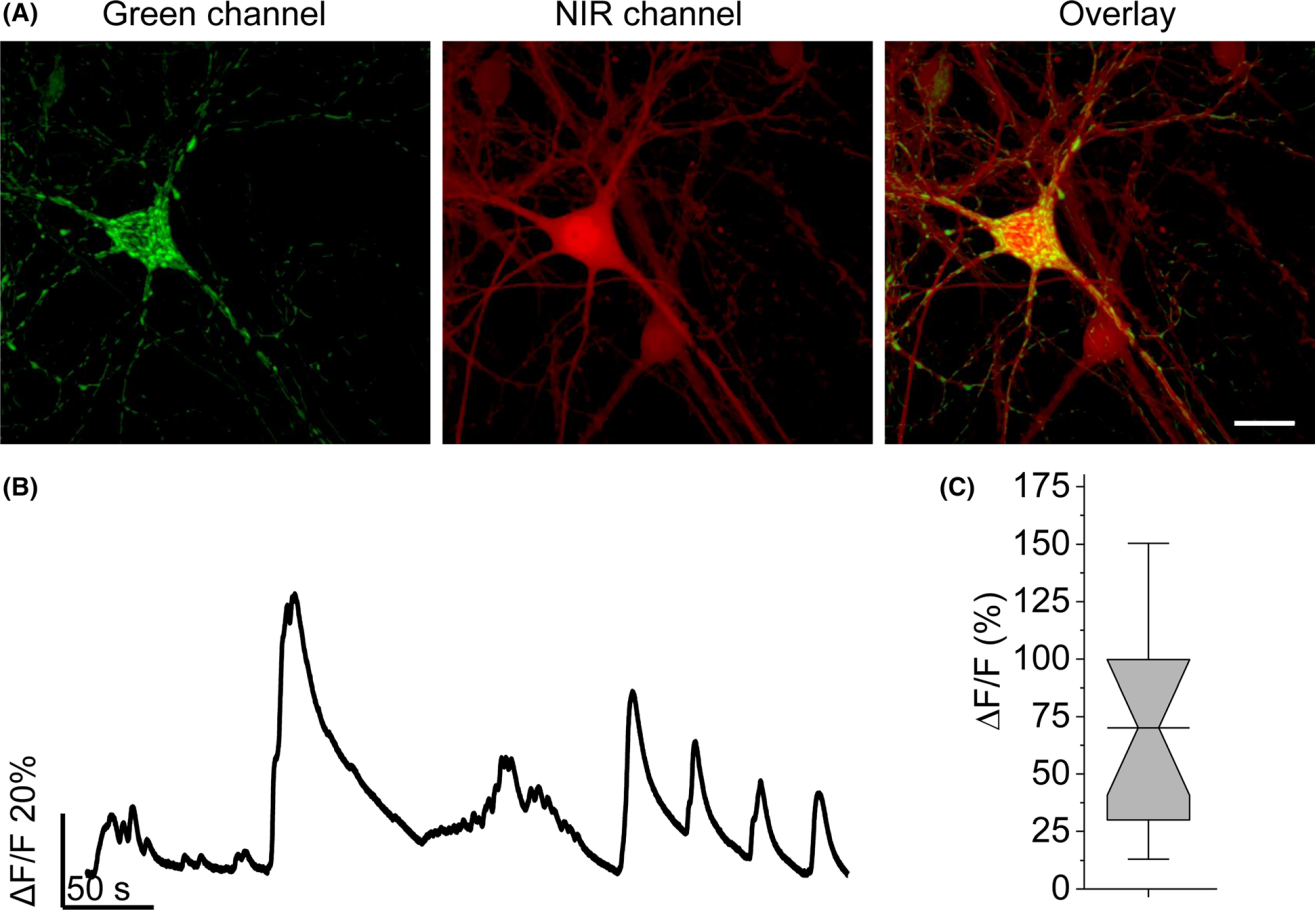
Validation of YTnC2 in mitochondria of cultured mouse neurons. (A) Representative fluorescence confocal images of neurons co‐expressing dMito‐YTnC2 and miRFP (*n* = 45 neurons from three independent transductions from one culture). Scale bar: 10 μm. (B) Representative single trial fluorescence trace of dMito‐YTnC2 signal in neuron during nonspecific activity monitored using widefield microscope (*n* = 14 neurons from three independent transductions from one culture). (C) Maximal Δ*F*/*F* for the experiment of B (*n* = 14 neurons from three independent transductions from one culture).

Since the dMito‐YTnC2 fusion localized in mitochondria of HeLa cells and responded to calcium transients with a large Δ*F*/*F* response (see the previous section), we applied dMito‐YTnC2 for visualization of calcium transients in the mitochondria of neurons. On DIV 4–5, we co‐transduced neuronal cultures with rAAVs particles carrying rAAV‐DJ‐CAG‐dMito‐YTnC2 and rAAV8‐hSyn‐miRFP. On DIV 12–18, neuronal cultures were imaged using widefield and confocal microscopes. According to confocal images, dMito‐YTnC2 localized in the mitochondria of neurons (Fig. 6A). Functional imaging of the neuronal cultures expressing the dMito‐YTnC2 fusion using a widefield microscope revealed spontaneous mitochondrial calcium activity of neurons with averaged Δ*F*/*F* response value of 0.70 ± 0.25 (Fig. 6B,C). Hence, dMito‐YTnC2 successfully visualized the mitochondrial nonspecific activity of neuronal cultures.

### Crystal structure of the YTnC2‐5 indicator in the presence of calcium ions

**Fig. 7:**
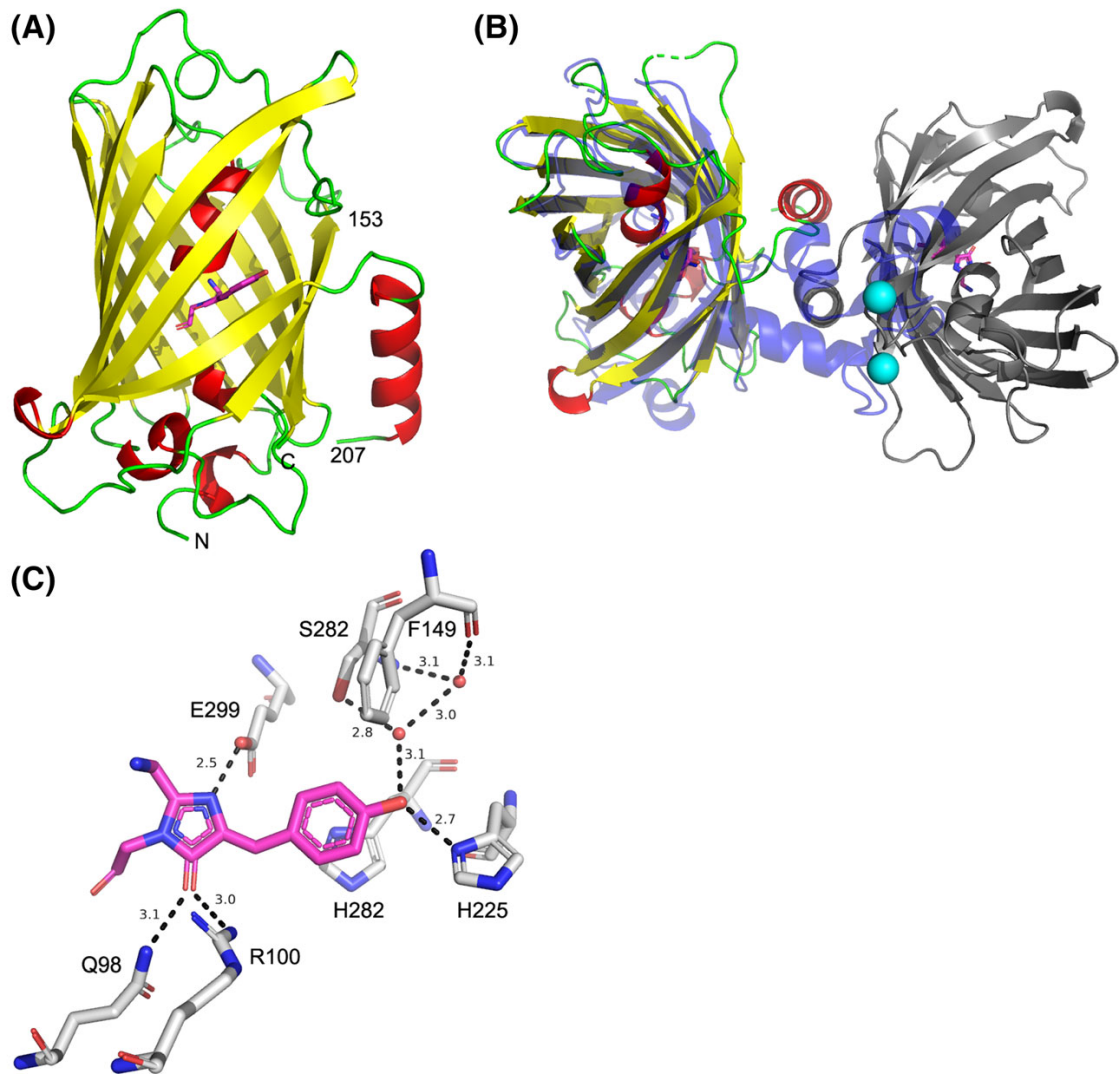
Crystal structure of YTnC2‐5. (A) Protein monomer is colored in accordance with secondary structure. Chromophore is shown in pink. Border residues are numbered (B) NTnC monomer (PDB ID—5MWC, blue, semitransparent) superposed on one of two YTnC2‐5 monomers from the asymmetric unit (colored as on panel A). The second YTnC2‐5 monomer from the asymmetric unit is colored in gray. Calcium ion from the NTnC structure is colored in cyan. (C) Environment of the YTnC2‐5 chromophore.

To understand, how the chromophore environment influenced the properties of the YTnC2 indicator, we solved the crystal structure of its mutant, YTnC2‐5 at 1.95 Å resolution (Table S3 and Fig. 7). The YTnC2‐5 variant had a two‐domain architecture with the covalently linked fluorescent and Ca^2+^‐binding domains (Fig. 2A). There were two almost identical YTnC2‐5 molecules in the asymmetric unit of the crystal. The structure of the YTnC2‐5 fluorescent part had a typical β‐barrel fold with the ^69^GYG^71^ chromophore located in the middle of the central α‐helix. In both molecules, almost the whole Ca^2+^‐binding domain (residues 154–206) had no electron density hindering its modeling (Fig. 7A). Based on crystal packing analysis, we could suggest that conformation of calcium‐binding module in the YTnC2‐5 structure differed from that in the structure of NTnC (PDB ID—5MWC) (Fig. 7B).

We therefore analyzed the environment of the chromophore (Fig. 7C). The chromophore had a *cis*‐configuration and formed five direct hydrogen bonds with Q98, R100, H225, and E299 residues and water‐mediated H‐bond with side chain of S282 residue. The tyrosine moiety of the chromophore was stacked with the H280 side chain. In addition, the OH‐group of the chromophore formed two H‐bonds with the main chain of F149 and S282 through two water molecules. F149 was the first amino acid residue from Linker 1 between the fluorescent part and the Ca^2+^‐binding Troponin C. We suggested that calcium ion‐induced conformational changes in the Ca^2+^‐binding part might be translated into the chromophore's fluorescence changes altering the hydrogen bond between F149 and the OH‐group of the chromophore.

Next, in the Ca^2+^‐bound fluorescent state of the YTnC2‐5, the OH‐group of the chromophore formed three H‐bonds with the neighboring residues, mentioned above. On the contrary, in the Ca^2+^‐bound dark state, the chromophore of the NTnC calcium indicator with the inverted phenotype formed only one H‐bond between the tyrosine moiety of the chromophore and R280 residue [10]. Hence, we could speculate that the chromophore of the YTnC2‐5 requires more tight anchoring in the fluorescent state to ensure rigidity of the chromophore compared with a more flexible chromophore in the nonfluorescent state.

## Conclusions

In conclusion, using directed molecular evolution in bacterial system, we developed enhanced version of Troponin C‐based GECI, YTnC2, which had superior characteristics *in vitro*, in the cytosol and mitochondria of mammalian cells and in cultured neurons over other Troponin C‐based GECIs.

Using FRAP experiments in the cytosol of mammalian cells, we demonstrated that YTnC2 practically did not interact with intracellular environment at physiological calcium concentrations and revealed just minor interactions at elevated calcium ions levels. Truncated version of the GCaMP6s indicator, called GCaM6s, with deleted M13‐like peptide revealed strong interactions with intracellular environment at elevated calcium ions levels [21]. Hence, these data support hypothesis that, in contrast to calmodulin, truncated Troponin C does not interact with cytosolic environment of mammalian cells.

Crystal structure of fluorescent domain of YTnC2‐5 indicator revealed chromophore's surrounding and allowed suggesting the mechanism of translation of conformational changes in Troponin C domain induced by calcium ions binding into fluorescent signal through altering the hydrogen bond between F149 and the OH‐group of the chromophore.

During the development of the YTnC2 enhanced version of the YTnC indicator in terms of brightness and dynamic range, we in parallel tried to enhance the dynamic range of the NTnC indicator. As a result, we found its enhanced version, called cNTnC. As compared to cNTnC, the YTnC2 indicator has 1.3‐fold lower molecular brightness, but 2.7‐fold faster calcium dissociation kinetics and 1.9‐fold larger Δ*F*/*F* response to APs in neurons. In contrast to YTnC2, the affinity of cNTnC to calcium ions dramatically in 8‐fold decreased in the presence of 1 mm Mg^2+^ ions, which are present in the cytosol of mammalian cells. In conclusion, the YTnC2 is currently the best Troponin C‐based green calcium indicator optimized for visualization of calcium neuronal activity both in the cytosol and lumen of mitochondria.

## Conflict of interest

The authors declare no conflict of interest.

### Peer review

The peer review history for this article is available at https://www.webofscience.com/api/gateway/wos/peer‐review/10.1002/2211‐5463.13702.

## Author contributions

OMS and FVS developed YTnC2 and characterized YTnC2, YTnC2‐5 *in vitro*, and mammalian cells. AYN and KMB performed crystallization of YTnC2‐5, data collection, and structural studies. AVV, YKA, and MVP performed protein purification. AMV performed stopped‐flow experiments. OVP made FRAP experiments. KDP characterized dMito‐YTnC2 in neurons. OMS, AMV, KMB, KDP, and FVS wrote the manuscript. All authors reviewed the manuscript.

## Supporting information


**Fig. S1.** Nucleotide sequence of the YTnC2 protein.
**Fig. S2.** Alignment of the amino acid sequences for the YTnC2, its YTnC2‐5 mutant, and its progenitor YTnC calcium indicators.
**Fig. S3.** Response of the YTnC2 indicator to thapsigargin‐induced Ca^2+^ variations in the lumen of mitochondria of the HeLa cells.
**Table S1.** List of primers.
**Table S2.** *In vitro* properties of YTnC2‐5 variant with higher affinity to calcium ions.
**Table S3.** Data collection, processing, and refinement.

## Data Availability

All the data generated and analyzed in the study are included in the article and its Supporting Information.
