# Supplementary material for: YTnC2, an improved genetically encoded green calcium indicator based on toadfish troponin C
Source: FEBS Open Bio. 2023 Sep 20;13(11):2047–60. doi: 10.1002/2211-5463.13702 (PMC10626279; doi:10.1002/2211-5463.13702)
Supplement: Supplementary file 1 — Fig. S1. Nucleotide sequence of the YTnC2 protein. Fig. S2. Alignment of the amino acid sequences for the YTnC2, its YTnC2‐5 mutant, and its progenitor YTnC calcium indicators. Fig. S3. Response of the YTnC2 indicator to thapsigargin‐induced Ca2+ variations in the lumen of mitochondria of the HeLa cells. Table S1. List of primers. Table S2. In vitro properties of YTnC2‐5 variant with higher affinity to calcium ions. Table S3. Data collection, processing, and refinement. [file FEB4-13-2047-s001.docx]

## YTnC2, an improved genetically-encoded green calcium indicator based on toadfish troponin C

Oksana M. Subach, Anna V. Vlaskina, Yulia K. Agapova, Alena Y. Nikolaeva, Anna M. Varizhuk, Podgorny O.V., Kiryl D. Piatkevich, Maxim V. Patrushev, Konstantin M. Boyko and Fedor V. Subach

**Supplementary Information**

**Supplementary Table S1 2**

**Supplementary Table S2 3**

**Supplementary Table S3 4**

**Supplementary Figure S1 6**

**Supplementary Figure S2 7**

**Supplementary Figure S3 8**

**Supplementary Table S1.** List of primers.

| **Primer** | **Primer sequence (5’-3’)** |
| --- | --- |
| **Fw-YFP-BglII** | gacAGATCTATGGTGAGCAAGGGCGAGGAG |
| **Rv-YFP-EcoRI** | GTCGAATTCttacttgtacagctcgtccatgcc |
| **BamHI-NES-mCher** | GGG GAT CCG ACA CCA TGC TTC AAC TTC CTC CTC TTG AAC GTC TTA CTC TTT CGA GAT CTG CTA GCC TCG AGA TGG TG |
| **mCher-BsrGI-r2** | ACTTGTACATTACTTATACAGCTCGTCCATGCCGCCGGTG |
| **Mito-KpnI** | ataggatccggtaccgccaccATGTCCGTCCTGACGCC |
| **Mito-BamHI** | ataggatccaccggtgacaccATGTCCGTCCTGACGCC |
| **Mito-NheI-r** | GAGGCTAGCAGATCTTGCAGGCCCCAACGAATG |
| **YTnC-NheI** | cgagatctgctagcATGGTGAGCAAGGGCGAG |
| **GCaMP-NheI** | cgagatctgctagcATGGTCGACTCATCACGTC |
| **TnC-EcoRI-HindIII-r** | AGA AAG CTT GAA TTC TTA CTT ATA CAG CTC GTC CAT G |
| **GCaMP-EcoRI-HindIII-r** | GTC AAG CTT GAA TTC CTA CTT CGC TGT CAT CAT TTG |
| **TnC-EcoRI-HindIII-r** | GTC AAG CTT GAA TTC TTA CTT ATA CAG CTC GTC CAT G |
| **Neon-EcoRI-rv3** | TCG GAA TTC CTT ATA CAG CTC GTC CAT G |
| **YTnC-AgeI** | taaccggtgacaccATGGTGAGCAAGGGCGAG |
| **GCaMP-AgeI** | taaccggtgacaccATGGTCGACTCATCACGTC |
| **GCaMP-EcoRI-r2** | cagGAATTCCTTCGCTGTCATCATTTG |

**Supplementary Table S2.** *In vitro* properties of the YTnC2-5 variant with higher affinity to calcium ions.

| **Properties** | | **YTnC2-5** | |
| --- | --- | --- | --- |
|  |  | **apo** | **sat** |
| **Absorbance maxima (nm)** | | 412 | 496 |
| **Emission maxima (nm)** | | 506 | 518 |
| **Quantum yield ^a^** | | 0.077 ± 0.01 | 0.54 ± 0.03 |
| **ε (mM^-1^ cm^-1^) ^b^** | | 49.6 ± 2.4 | 63.9 ± 2.1 |
| **Brightness vs mEGFP (%) ^c^** | | 11.9 | 109 |
| **ΔF/F** | **0 mM Mg^2+^** | 41 ± 4 | |
|  | **1 mM Mg^2+^** | 16.1 ± 0.3 | |
| **p*K*_a_** | | 4.96 ± 0.03  6.25 ± 0.05 | 5.44 ± 0.01 |
| **K_d_ (nM) ^d^** | **0 mM Mg^2+^** | 218 ± 4 [n=1.74 ± 0.06] | |
|  | **1 mM Mg^2+^** | 227 ± 5 [n=1.50 ± 0.05] | |
| **k_obs_ (s^-1^) ^e^** | **k_1_ (contrib., %)** | 1.3 ± 0.1 (39 ± 4) | |
|  | **k_2_ (contrib., %)** | 0.5 ± 0.1 (61 ± 4) | |
| **k_off_ (s^-1^) ^f^** | | 0.30 ± 0.01 | |
| **t_1/2_^off^ (s) ^g^** | | 2.4 ± 0.1 | |

^a^ Quantum yields (QYs) were determined at pH 7.20. mEGFP (QY=0.60 [18]) and mTagBFP2 (QY=0.64 [19]) were used as reference standards for 496- and 412-nm absorbing states, respectively. ^b^ Extinction coefficient (ε) was determined by alkaline denaturation; mEGFP in 1xPBS buffer had ε = 53.3±3.6 mM^-1^ cm^-1^. ^c^ Brightness was calculated as a product of the QY and ε. ^d^ Hill coefficient is shown in square brackets. ^e^ The observed association rates were determined at 300 nM Ca^2+^ concentration. GCaMP6f at 300 nM Ca^2+^ concentration had k_obs_ value of 1.28 ± 0.01 sec^-1^. ^f^ k_off_ values were estimated from calcium dissociation curves at 1000 nM starting Ca^2+^-free concentration using mono or double exponential decay fitting with individual exponent contributions shown in the brackets. GCaMP6f had k_off_ value of 1.89 ± 0.07 s^-1^. ^g^ GCaMP6f had t_off_ value of 0.37±0.04 s.

**Supplementary Table 3.** Data collection, processing, and refinement.

| **Data Collection** | |
| --- | --- |
| Diffraction source | BL41XU, Spring8 |
| Wavelength (Å) | 1.0 |
| Temperature (K) | 100 |
| Detector | EIGER 16M |
| Crystal-to-detector distance (mm) | 280.0 |
| Rotation range per image (°) | 0.5 |
| Total rotation range (°) | 360 |
| Space group | P6_5_ |
| a, b, c (Å) | 65.74; 65.74; 315.04 |
| α, β, γ (°) | 90.0; 90.0; 120.0 |
| Unique reflections | 55880 (3979) |
| Resolution range (Å) | 46.14-1.95 (2.0-1.95) |
| Completeness (%) | 100 (100) |
| Average redundancy | 20.2 (19.1) |
| 〈*I*/σ(*I*)〉 | 6.5 (0.9) |
| Rpim (%) | 7.1 (83.0) |
| CC_1/2_ | 99.2 (46.8) |
| **Refinement** | |
| R_fact_ (%) | 18.3 |
| R_free._ (%) | 22.9 |
| Bonds (Å) | 0.01 |
| Angles (°) | 2.03 |
| **Ramachandran plot** |  |
| Most favored (%) | 97.3 |
| Allowed (%) | 2.5 |
| **No. atoms** |  |
| Protein | 3909 |
| Water | 214 |
| Chromophore | 38 |
| Other ligands | 14 |
| **B-factors (Å^2^)** |  |
| Protein | 31.28 |
| Water | 33.21 |
| Chromophore | 27.32 |
| Other ligands | 41.92 |
| Molprobity score | 1.77 |
| PDB ID | 8P6G |

Values in parenthesis are for the highest-resolution shell.

**YTnC2-stop gene:**

ATGGTGAGCAAGGGCGAGGAGCTGTTCACCGGGGTGGTGCCCATCCTGGTCGAGATGGTCGGCGACGTAAACGGCCATAGGTTCAGCGTGTCCGGCGAGGGCGAGGGCATTGCCACCTACGGCATGCTGACCCTGAAGCTCATCTGCACCACCGGCGAGCTGCCCGTGCCCTGGCCCACCCTCGTGACCACCCTCGGCTACGGCGTGGCGTGCTTCGCCCGCTACCCCGACCACATGAAGCAGCACGACTTCTTCAAGTCCGCCATGCCCGAGGGCTACGTCCAGGAACGCACCATCTTCTTCAAAGGCGACGGCTACTACAAGACCCGCGCCGAGGTGAAGTTCGAGGGCGACACCCTGGTGAACCGCATCGAGCTGAAGGGCTTCGACTTCAGGGAGGACGGCAACATCCTGGGTCACAAGCTGGGGTACAACTTCGACTTGAGCGAAGAAGAGCTAACAGAGTCCTTTCGCACCTTTGACAAGGATGGGGATGGTTTCATCGACAGGGAGGAATTTGGAGGCATCATCCGCCTTACTGGAGAACAGCTCACAGATGAGGATTCCGATGAGATTTTTGGAGACTCAGACACGGACAAAAATGGAAGGATTGACTTTGGTGAGTTCCTGAAGGTGGTGGAGAATGTCCGGGGCATCAACAGCCACAACGTCTATATCACCGCCGACAAGCAGAAGAACGGCGTCAAGGCCCACTTCGAGATCCGCCACAACCTCGAGGACGGCAGCGTGCAGCTCGCCGACCACTACCAGCTGAACACCCCCATCGGCGACGGCCCCGTGCTGCTGCCCGACAACCACTACCTGCGCCACCAGTCCGCCCTGAGCAAAGACCCCAACGAGAAGCGCGATCACATGGTCCTGCAGGAGTTCGTGACTGCCGCCGGGAtCACTCACGGCATGGACGAGCTGTACAAGTAA

**NES-YTnC2-NES3-stop gene:**

atgcttcaacttcctcctcttgaacgtcttactctttcgagatctatggtgagcaagggcgaggagctgttcaccggggtggtgcccatcctggtcgagatggtcggcgacgtaaacggccataggttcagcgtgtccggcgagggcgagggcattgccacctacggcatgctgaccctgaagctcatctgcaccaccggcgagctgcccgtgccctggcccaccctcgtgaccaccctcggctacggcgtggcgtgcttcgcccgctaccccgaccacatgaagcagcacgacttcttcaagtccgccatgcccgagggctacgtccaggaacgcaccatcttcttcaaaggcgacggctactacaagacccgcgccgaggtgaagttcgagggcgacaccctggtgaaccgcatcgagctgaagggcttcgacttcagggaggacggcaacatcctgggtcacaagctggggtacaacttcgacttgagcgaagaagagctaacagagtcctttcgcacctttgacaaggatggggatggtttcatcgacagggaggaatttggaggcatcatccgccttactggagaacagctcacagatgaggattccgatgagatttttggagactcagacacggacaaaaatggaaggattgactttggtgagttcctgaaggtggtggagaatgtccggggcatcaacagccacaacgtctatatcaccgccgacaagcagaagaacggcgtcaaggcccacttcgagatccgccacaacctcgaggacggcagcgtgcagctcgccgaccactaccagctgaacacccccatcggcgacggccccgtgctgctgcccgacaaccactacctgcgccaccagtccgccctgagcaaagaccccaacgagaagcgcgatcacatggtcctgcaggagttcgtgactgccgccgggatcactcacggcatggacgagctgtacaagcttgctcttaagttggctggacttgatattggttcttaa

dMito-YTnC2-stop gene:

atgtccgtcctgacgccgctgctgctgcggggcttgacaggctcggcccggcggctcccagtgccgcgcgccaagatccattcgttggggcctgcaagatccagtgtattaacacctctgctgctgcggggcttgacaggctcggcccggcggctcccagtgccgcgcgccaagattcacagtcttggtccagctagatctgctagcatggtgagcaagggcgaggagctgttcaccggggtggtgcccatcctggtcgagatggtcggcgacgtaaacggccataggttcagcgtgtccggcgagggcgagggcattgccacctacggcatgctgaccctgaagctcatctgcaccaccggcgagctgcccgtgccctggcccaccctcgtgaccaccctcggctacggcgtggcgtgcttcgcccgctaccccgaccacatgaagcagcacgacttcttcaagtccgccatgcccgagggctacgtccaggaacgcaccatcttcttcaaaggcgacggctactacaagacccgcgccgaggtgaagttcgagggcgacaccctggtgaaccgcatcgagctgaagggcttcgacttcagggaggacggcaacatcctgggtcacaagctggggtacaacttcgacttgagcgaagaagagctaacagagtcctttcgcacctttgacaaggatggggatggtttcatcgacagggaggaatttggaggcatcatccgccttactggagaacagctcacagatgaggattccgatgagatttttggagactcagacacggacaaaaatggaaggattgactttggtgagttcctgaaggtggtggagaatgtccggggcatcaacagccacaacgtctatatcaccgccgacaagcagaagaacggcgtcaaggcccacttcgagatccgccacaacctcgaggacggcagcgtgcagctcgccgaccactaccagctgaacacccccatcggcgacggccccgtgctgctgcccgacaaccactacctgcgccaccagtccgccctgagcaaagaccccaacgagaagcgcgatcacatggtcctgcaggagttcgtgactgccgccgggatcactcacggcatggacgagctgtacaagtaa

**Figure S1. Nucleotide sequences of the YTnC2 protein alone and in fusions.** NES and NES3 are nuclear export sequences. dMito is the mitochondrial presequence of human cytochrome c oxidase subunit VIII.

**
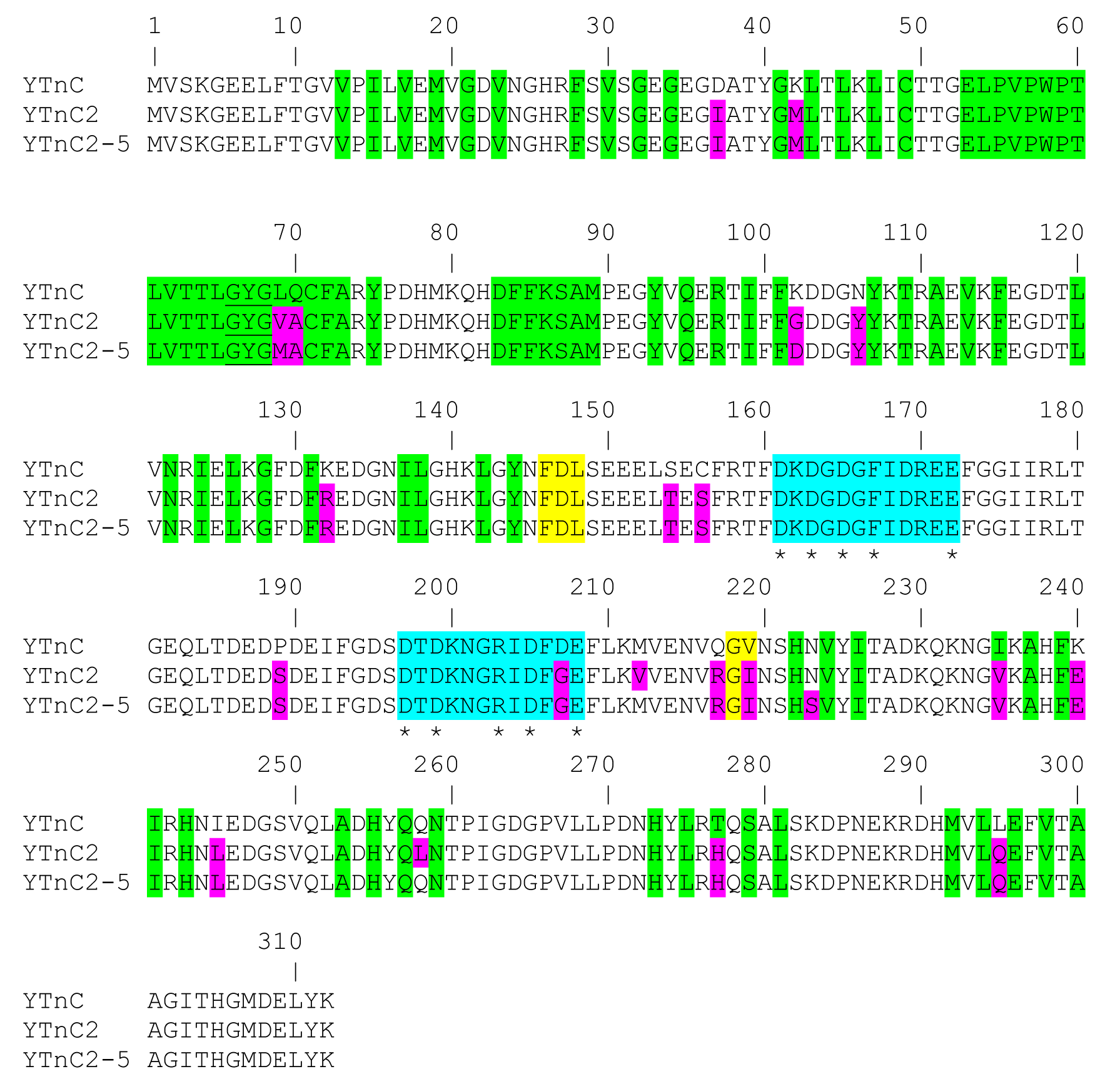
**

**Figure S2. Alignment of the amino acid sequences for the YTnC2, its YTnC2-5 mutant and its progenitor YTnC calcium indicators.** Residues from fluorescent part buried in β-can are highlighted with green. Residues that are forming chromophore are underlined. Calcium ions-coordinating residues are selected with asterisk according to X-ray structure of the NTnC indicator [10]. Residues that are forming Ca^2+^-binding loops are highlighted in blue. Mutations in YTnC2 and YTnC2-5 related to the original YTnC are highlighted in magenta. Linkers between truncated troponin C and fluorescent domain are in yellow.

**
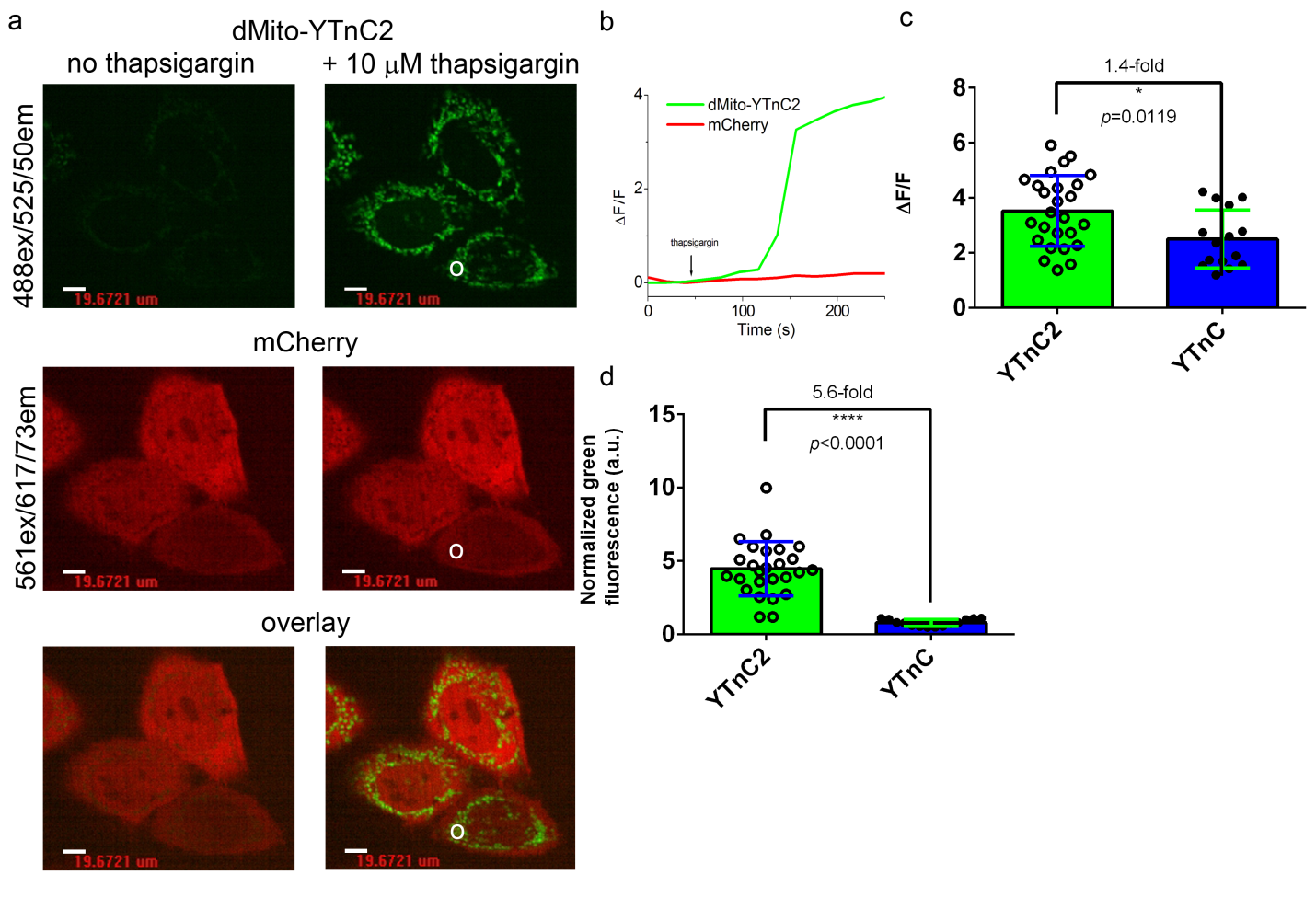
**

**Figure S3.** **Response of the YTnC2 indicator to thapsigargin induced Ca^2+^ variations in the lumen of mitochondria of the HeLa cells**. (a) Confocal images of HeLa cells co-expressing green dMito-YTnC2 calcium indicator and red mCherry RFP before and after addition of 10 µM thapsigargin. Scale bars, 20 µm. (b) The graph illustrates changes in green fluorescence of the dMito-YTnC2 and mCherry over time in response to the addition of 10 µM of thapsigargin (shown by arrow). The changes on the graph correspond to the area indicated with white circle on the panel a. (c) Averaged ΔF/F responses for the dMito-YTnC2 (n = 26 cells, two cultures) and dMito-YTnC (n = 15 cells, two cultures) indicators to the addition of the 10 µM of thapsigargin. (d) Averaged maximal brightness of the dMito-YTnC2 (n = 26 cells, two cultures) and dMito-YTnC (n = 15 cells, two cultures) indicators normalized to the red fluorescence of the mCherry RFP. To estimate the significance of the difference between two values, we used the Mann–Whitney rank sum test and provided p values calculated for the two-tailed hypothesis. Error bars represent the standard deviation. *, p-value is 0.01 to 0.05. ****, p-value is < 0.0001.
